# Improving search filter development: a study of palliative care literature

**DOI:** 10.1186/1472-6947-7-18

**Published:** 2007-06-28

**Authors:** Ruth M Sladek, Jennifer Tieman, David C Currow

**Affiliations:** 1 National Institute of Clinical Studies (NICS) PhD Scholar, Flinders University, Bedford Park, South Australia, Australia; 2 Knowledge Network, Department of Palliative and Supportive Services, Flinders University, Bedford Park, South Australia, Australia; 3 Department of Palliative and Supportive Services, Flinders University, Bedford Park, South Australia, Australia

## Abstract

**Background:**

It is difficult to systematically search for literature relevant to palliative care in general medical journals. A previously developed search filter for use on OVID Medline validated using a gold standard set of references identified through hand searching, achieved an unacceptably low sensitivity (45.4%). Retrieving relevant literature is integral to support evidence based practice, and understanding the nature of the incorrectly excluded citations (false negatives) using the filter may lead to improvement in the filter's performance.

**Methods:**

The objectives were to describe the nature of subjects reflected in the false negative citations and to empirically improve the sensitivity of the search filter. A thematic analysis of MeSH terms by three independent reviewers was used to describe the subject coverage of the missed records. Using a frequency analysis of MeSH terms, those headings which could individually contribute at least 2.5% to sensitivity (occurring 19 or more times) were added to the search filter. All previously run searches were rerun at the same time as the revised filter, and results compared.

**Results:**

Thematic analysis of MeSH terms identified thirteen themes reflected in the missing records, none of them intrinsically palliative. The addition of six MeSH terms to the existing search filter (physician-patient relations, prognosis, quality of life, survival rate, treatment outcome and attitude to health) led to an increase in sensitivity from 46.3% to 64.7%, offset by a decrease in precision from 72.6% to 21.9%.

**Conclusion:**

The filter's sensitivity was successfully increased using frequency analysis of MeSH terms, offset by a decrease in precision. A thematic analysis of MeSH terms for the false negative citations confirmed the absence of any intrinsically palliative theme or term, suggesting that future improvements to search filters for palliative care literature will first depend on better identifying how clinicians and researchers conceptualise palliative care. It is suggested that a constellation of parameters: stage of disease (advanced or active), prospect of cure (little or none), and treatment goals (primarily quality of life) may ultimately inform search strategies. This may be similarly true for chronic diseases, which share the inherent passage of time which marks them apart from acute, and therefore more readily identifiable, episodes of care.

## Background

It is difficult to systematically search for literature relevant to palliative care. It is a diffuse subject, embracing topics from multiple other disciplines, and with relevant papers likely to be published in general medical journals as well as discipline specific journals. To facilitate improved identification of these papers, an earlier study formulated and evaluated palliative care search filters for use in the general medical literature, as part of a larger project, the Evidence Based (CareSearch) Project [1,2]. This paper presents an overview of the previous study, and reports findings from current research which investigates further improvements to filter performance.

### The Original Study [1]

Using a methodological approach often used to evaluate new diagnostic tests in medicine, and previously used to develop validated search filters [3], four general medical journals were hand-searched to identify articles relevant to palliative care, forming a 'gold standard' reference set of 773 relevant citations. The journals chosen (JAMA, BMJ, The Lancet and Annals of Internal Medicine) were selected for their wide availability, balance between North American and European perspectives, established reputations and underlying peer review processes. Searches comprising MeSH terms and textwords were created for use in OVID Medline and retrieved references were compared to the gold standard reference set. Sensitivity, specificity, precision and accuracy rates were calculated (Table 1). The best performing search (combining 9 MeSH terms and 3 textwords with the Boolean OR operator) referred to as the Master Search (Table 2), originally achieved a sensitivity of 45.4%. In other words, a noteworthy 54.6% of the citations we knew were in the gold standard reference set and which contained information relevant to palliative care, failed to be retrieved using the search strategy. Given a key implication of missing citations may be that it contributes to bias in systematic reviews [4], we concluded that analysis of the missed citations might fruitfully yield further MeSH terms which could be used to improve the sensitivity of the search.

**Table 1:** Rate definitions (sensitivity, specificity, accuracy, precision)

	**Gold Standard Reference Set**	
	Relevant	Not Relevant
	
**Medline Result**		
Articles retrieved	a (correct inclusion)	b (incorrect inclusion)
Articles not retrieved	c (incorrect exclusion)	d (correct exclusion)

Sensitivity = a/(a+c); specificity = d/(b+d); precision = a/(a+b); accuracy = (a+d)/(a+b+c+d)

**Table 2:** Master Search Strategy

exp advance care planning/or
exp attitude to death/or
exp bereavement/or
death/or
hospices/or
life support care/or
palliative care/or
terminal care/or
terminally ill/or
palliat$.tw. or
hospice$.tw. or
terminal care.tw.

This current paper reports a study investigating the nature of this subset of records. The objectives were to improve empirically the sensitivity of the Master Search and to describe the nature of subjects reflected in the incorrectly excluded citations.

## Methods

This research was undertaken May – November 2006 at the Repatriation General Hospital, Adelaide, South Australia. A set of incorrectly excluded citations was created by comparing records in the gold standard reference set (those identified in the previous study using a hand search) with records retrieved by the Master Search (the best performing MeSH and textword search). Items held in the Gold Set but not retrieved by the Master Search were regarded as false negatives, that is, they were incorrectly excluded when the Master Search was run. A set of 418 records were identified, and was called the Silver Set to distinguish it from the original hand search set of 773 records (the Gold Set). An Excel spreadsheet containing the citation details of the incorrectly excluded items was created.

### Frequency Analysis & Revised Master Search

A frequency analysis of all MeSH terms in the Silver Set was undertaken. These were originally derived from four general medical journals; JAMA, The Lancet, BMJ and Annals of Internal Medicine, published from 1999 to 2001. The entire set was downloaded into a Word document, and all data except for MeSH terms were deleted. These terms were reformatted into a list, which was then exported into an Excel spreadsheet, and sorted into alphabetical order. Frequencies were tallied for all terms, including sub headings attached to MeSH terms, but ignoring whether or not terms were tagged as a major heading.

A Revised Master Search was devised incorporating the most frequently occurring MeSH terms. It was calculated a priori that an increase of 2.5% sensitivity in the Gold Set (n = 773) would equate to 19 additional records being retrieved from the Silver Set. This was a subjective estimate of the percentage increase that might represent a worthwhile improvement when offset against anticipated decline in precision. The Revised Master Search therefore combined the previous Master Search with the additional MeSH terms which demonstrated frequencies of higher than 19 (using the Boolean operator 'or'). Retrieved citations were then compared with those in the Gold Set, using a purposely written computer program called SSV (Search Strategy Validation), described elsewhere [1]. It is noted that due to the dynamic nature of the OVID Medline database over time (such as changes to MeSH indexing and retrospective indexing), it was necessary to re-run all previous searches in addition to the Revised Master Search to ensure accurate comparison of results. These searches included three previously published (yet not validated) search strategies relevant to palliative care [5-7], which we used to compare rates; the published Cochrane PAPAS Review Group Search Strategy, and strategies used by the National Institute for Health and Clinical Excellence (NICE) and the Scottish Intercollegiate Guidelines Network (SIGN) in the development of their respective guidelines. All such searching was undertaken on 10^th ^November 2006.

### Thematic Analysis

As MeSH terms should reflect the major subject foci of an indexed article, the full list of all terms used was provided to RS (medical librarian), JT (researcher in palliative care) and DC (senior palliative care medical consultant). Each separately, and independently, identified the major themes they thought were reflected by the range of these terms. One list (DC's) was tabled in a spreadsheet, and RS and JT then collocated their identified themes against this list according to similarity. For example, *drug related therapies *(RS) and *palliative medications *(JT) were grouped with *therapeutics for symptoms *(DC) using this process. A meeting was then held during which the spreadsheet was discussed, and consensus reached regarding a list of broad themes.

## Results

Only minor changes were noted to the overall reported sensitivity, specificity, precision and accuracy rates for the different searches undertaken at 10^th ^November 2006 compared to 30^th ^June 2005. (Table 3).

**Table 3:** Comparisons of Master Search and modified PAPAS, NICE and SIGN searches: 30^th ^June 2005 versus 10^th ^November 2006

	**Sensitivity**		**Specificity**		**Precision**		**Accuracy**	
	**June 2005**	**Nov 2006**	**June 2005**	**Nov 2006**	**June 2005**	**Nov 2006**	**June 2005**	**Nov 2006**
**Master Search**	45.4%	46.3%	99.4%	99.45%	73.0%	72.6%	97.4%	97.4%
**PAPAS**	45.2%	45.9%	99.3%	99.3%	71.4%	72.0%	97.3%	97.4%
**NICE**	40.6%	41.0%	95.6%	95.9%	26.4%	26.4%	93.5%	94.0%
**SIGN**	56.9%	59.1%	92.1%	92.6%	22.0%	21.9%	90.8%	92.0%

### Frequency Analysis

1873 unique MeSH terms were used a total of 4293 occasions, meaning that each false negative citation was indexed with a mean of 4.48 unique MeSH terms (1873 MeSH terms/418 references) and a total of 10.27 MeSH terms per article (4293 occasions/418 references). When certain terms were disregarded (age groups, human/animal, gender, countries and research designs such as randomized controlled trials), the most frequently occurring terms were *physician-patient relations *(39), *prognosis *(29), *quality of life *(26), *survival rate *(26), *treatment outcome *(23), and *attitude to health *(21) (Table 4).

**Table 4:** Most Frequently Used MeSH Terms (> 10 citations)

**MeSH Term**	**Frequency**
**>30 Occurrences**	
Physician-patient relations	39
**>20 Occurrences**	
Prognosis	29
Quality of Life	26
Survival Rate	26
Treatment Outcome	23
Attitude to Health	21
**>10 Occurrences**	
Chronic Disease	18
Survival Analysis	18
Pain/pathophysiology	16
Risk Assessment	16
Neoplasms/therapy	15
Adaptation, Psychological	14
Breast Neoplasms/psychology	14
Pain/therapy	13
Practice Guidelines	13
Time Factors	13
Antineoplastic Agents/therapeutic use	12
Antineoplastic Combined Chemotherapy Protocols/therapeutic use	12
Breast neoplasms/mortality	12
Combined Modality Therapy	12
Ethics, Medical	12
Patient Satisfaction	12
Quality of Health Care	12
Age Factors	11
Communication	11
Pain/etiology	11
Risk Factors	11

When these six MeSH terms were added to the Master Search to create the Revised Master Search (Table 5) and results compared to the Gold Set, sensitivity increased from 46.3% to 64.7% (+ 18.4%), whilst specificity decreased from 99.4% to 92.0% (- 7.4%). Precision decreased from 72.6% to 21.9% (-50.7%) (Table 6).

**Table 5:** Revised Master Search Strategy

exp advance care planning/or
exp attitude to death/or
exp bereavement/or
death/or
hospices/or
life support care/or
palliative care/or
terminal care/or
terminally ill/or
palliat$.tw. or
hospice$.tw. or
terminal care.tw. or
physician-patient relations/or
prognosis/or
quality of life/or
survival rate/or
treatment outcomes/or
attitude to health/

**Table 6:** Comparison of Master Search with stepwise addition of each additional MeSH term

	**Sensitivity**	**Specificity**	**Precision**	**Accuracy**
Master Search	46.3%	99.4%	72.6%	97.4%
Master Search OR physician-patient relations/	51.8%	97.8%	45.9%	96.1%
Master Search OR physician-patient relations/OR prognosis/	55.0%	96.5%	36.5%	95.1%
Master Search OR physician-patient relations/OR prognosis/OR quality of life/	58.6%	96.0%	34.6%	94.7%
Master Search OR physician-patient relations/OR prognosis/OR quality of life/OR survival rate/	61.6%	95.1%	31.2%	94.0%
Master Search OR physician-patient relations/ OR prognosis/OR quality of life/OR survival rate/OR treatment outcome/	63.8%	92.6%	23.2%	91.6%
Master Search OR physician-patient relations/OR prognosis/OR quality of life/OR survival rate/OR treatment outcome/OR attitude to health/(= Revised Master Search)	64.7%	92.0%	21.9%	91.1%

### Thematic Analysis

Each researcher identified slightly different individual themes in their individual analyses (DC = 9, RS = 23, JT = 23). Consensus was readily achieved in discussion, resulting in a final list of 13 broad themes, for example, drug therapies for symptoms, non-drug therapies for symptoms, disease modifying therapies, and pain and other symptoms (Table 7).

**Table 7:** Themes reflected in MeSH terms

Diseases
Ethical issues
Existential issues
Health Professional Issues
Organisational Issues
Pain and Other Symptoms
Patient Issues
Patient-Professional Relationship
Psychology, Communication, Attitudes
Quality of Life
Therapies – Drug for Symptoms
Therapies – Non drug for Symptoms
Therapies – Disease Modifying

## Discussion

Incorrectly excluded citations from a previously validated palliative search filter were studied using frequency analysis of MeSH terms, resulting in the additional inclusion of headings which empirically improved the sensitivity of the original Master Search. Not unexpectedly, both specificity and precision decreased with this Revised Master Search.

Notably, the sensitivity of the Revised Master Search was higher than that achieved by three relevant and published search strategies [5-7], modified slightly for comparison purposes in our early study. Previously it was found that the SIGN strategy had the highest sensitivity (56.9%), although this came at a cost of substantially lower precision (22.0%). The Revised Master Search has achieved greater sensitivity (64.7% compared to 59.1%) than the SIGN strategy, and similar specificity (92.0% compared to 92.6%). Both have equal precision, namely a low 21.9%. This is not insignificant as in real terms it means that 1784 of the retrieved citations were irrelevant. The original Master Search may still represent the best overall compromise between sensitivity and precision, however, for those requiring improved sensitivity, the Revised Master Search represents the best strategy.

The overall large number of unique MeSH terms supports the conceptualization of information relevant to palliative care as diffuse, that is, spanning many subject areas [1]. No clearly identifiable palliative terms (for example 'hospice' is readily recognized as a relevant term) were shown to be inadvertently omitted from the original Master search. However the issue under consideration in this study, is really the identification of contexts which make the incorrectly excluded articles relevant to palliative care, given they were not indexed with the readily identifiable relevant MeSH terms. If the authors didn't identify those MeSH terms as relevant, and neither did the Medline indexers, why should a palliative care clinician and researcher identify those articles as relevant to palliative care?

The thematic analysis of all MeSH terms included in the Silver Set (incorrect exclusions from the first study) was used to investigate this question, and yet did not provide any clear answers. None of the themes, or the individual MeSH terms which we reviewed to identify the themes, are intrinsically 'palliative'. Indeed, a similar analysis in any number of other specialties, such as rheumatology or internal medicine, might reveal a similar range of themes. For example, the themes of *ethics, pain and other symptoms*, and *drug therapies for symptoms *are relevant to many other disciplines, not just palliative care.

It is true that summarizing the Silver Set using themes might obscure MeSH terms which are more readily identifiable as palliative, however this was not the case. Using the 'diseases' theme as an example, some of the MeSH terms which led to the identification of this theme included: *arthritis, rheumatoid; cardiovascular diseases; depression; dementia; *and *asthma*. In isolation, none of these diseases are necessarily palliative. Numerous terms relating to cancer were also revealed, for example: *adenocarcinoma*; *breast neoplasms; brain neoplasms; prostatic neoplasms; *and *neoplasms*. Although many cancers are progressively life limiting, the prognosis for many cancers, particularly with early intervention, is good. Thus it cannot be presumed that these are inherently relevant.

If the MeSH terms individually do not suggest immediate relevance to palliative care, then it is perhaps the underlying constellation of concepts and terms in individual articles which led to their original identification. For example, perhaps a cluster of MeSH terms and textwords, in unison, reflect something which a clinician recognizes as relevant to palliative care. This would not be surprising, as it would likely reflect the multifaceted nature of what constitutes an episode of palliative care. In developing such a definition for the Australian National Sub-acute and Non-acute Patient (AN-SNAP) Version 1 Casemix Classification, a cluster of parameters identified an episode as palliative, rather than simply diagnosis. These parameters included stage of disease (advanced or active), prospect of cure (little or none), and treatment goals (primarily quality of life), evidenced by assessment and management of a range of individual needs (physical, psychosocial, emotional and spiritual), and a grief and bereavement process for the individual and their carers/family [8].

We also speculate that it is the changing nature of a disease over time which can identify it as potentially 'palliative'. All of the six additional MeSH terms incorporated in the Revised Master Search intrinsically reflect the passage of time. This is most identifiable with *prognosis*, *survival rate *and *treatment outcome*. Yet even for *physician-patient relations*, *quality of life*, and *attitude to health*, all reflect a notion of an inherent timeline.

This study highlights the value of frequency analysis of MeSH terms, a third generation approach considered to be a more rigorous method [9]. Whilst we still had a subjective cut-off point, the selection of included additional MeSH terms was based on an objective and replicable process. The overall sensitivity of the search was successfully increased by the addition of terms identified through the quantitative approach of frequency analysis. The palliative care filter has limitations as outlined in our original report [1]. However, given that high sensitivity is desirable for systematic reviews of the literature, this revised strategy represents an improved search in this regard. The original Master Search, however, still remains the best compromise between sensitivity, specificity and precision for clinicians.

One limitation of this study relates to the online version OVID Medline not being static due to updating over time. Changes were made to the underlying records in between the original study and the current research (approximately 18 months). Whilst this was addressed by re-running all searches at the later date for comparison purposes, it is true that the actual MeSH indexing may be different to the original study from which the Silver Set was created. This means that the underlying frequencies of MeSH terms used to inform the revision of the search may be marginally inaccurate. We do not think the implications are great however, as our original study located 351 records which were in both the Gold Set and the Master Search; when this search was rerun the figure rose to 358 records. In other words, there were an additional seven relevant records retrieved. Given the publication period chosen for the original study was 1999 to 2001, it is sobering to realise that retrospective indexing has so recently been undertaken, that is, in 2006. Such delays in indexing may have implications when identifying the literature for systematic reviews, depending on the topic, and support the need for search strategies that are not solely contingent upon searching bibliographic databases.

Generalisability in this study is difficult to assess. Because it builds on the previous study, it shares issues such as being based on a restricted subset of journals, described more fully elsewhere [1]. No validation set of records was used to test MeSH terms, and terms were selected on the bases of frequency, hence none were added that demonstrated high precision. Although the six most frequently appearing MeSH terms did not actually have any subheadings, the analysis was undertaken using exact MeSH terms (with or without subheadings) as they appeared in the citations. However, database limitations mean it is not possible to search for MeSH terms without subheadings; a searcher has only two choices – to select all subheadings or to select specific subheadings. We chose all subheadings, as this preserved the intent of a frequency analysis and would ensure that all records tagged with the six most frequent MeSH terms would be retrieved. However the limitation is that sensitivity may be overestimated (other less frequent MeSH terms with subheadings may be included) and precision and accuracy underestimated (additional records will contribute to the denominator of those rates). Given the focus of this research was to improve sensitivity, we explored this possibility of bias and note that only one citation relating to the six MeSH terms had a subheading (quality of life/psychology). Our sensitivity rate therefore, is held to be accurate.

Whilst this study has described the themes represented by references incorrectly excluded in the use of a validated search strategy, it would be inappropriate to suggest these themes reflect how palliative care overall is conceptualized. A similar analysis of 'correctly included' references, that is, those which were readily identified as relevant to palliative care, would also be required. The most important next step may be an exploration of clusters of MeSH terms and concepts, which together reflect that an article is likely relevant to palliative care.

## Conclusion

The sensitivity of an existing search filter for identifying literature relevant to palliative care was successfully increased from 46.3% to 64.7% using frequency analysis of MeSH terms. This was notably offset by a decrease in precision from 72.6% to 21.9%. A thematic analysis of MeSH terms for incorrectly excluded references confirmed the absence of any single theme (or term) that was intrinsically palliative, suggesting that future improvements to search filters for literature relevant to palliative care literature will first depend on better identifying how clinicians and researchers conceptualise this discipline. On the basis of an existing definition for a palliative care episode of care, we suggest it may be a constellation of parameters: stage of disease (advanced or active), prospect of cure (little or none), and treatment goals (primarily quality of life) which may ultimately inform improved searches. This may be similarly true for chronic diseases, which share the inherent passage of time which marks them apart from acute, and therefore more readily identifiable, episodes of care.

## Competing interests

The author(s) declare that they have no competing interests.

## Authors' contributions

RS, JT and DC participated in the conception and design of the study. JT identified and extracted the records referred to as the Silver Set. RS undertook the frequency analysis of MeSH terms. All authors undertook the thematic analysis of MeSH terms. RS, JT and DC discussed the findings and the implications of same, and RS drafted the manuscript to reflect that discussion. All authors were involvedin the revision of paper and authorised the final version for submission.

## Pre-publication history

The pre-publication history for this paper can be accessed here:


